# Longitudinal association of dietary acid load with kidney function decline in an older adult population with metabolic syndrome

**DOI:** 10.3389/fnut.2022.986190

**Published:** 2022-09-30

**Authors:** Cristina Valle-Hita, Nerea Becerra-Tomás, Andrés Díaz-López, Zenaida Vázquez-Ruiz, Isabel Megías, Dolores Corella, Albert Goday, J. Alfredo Martínez, Ángel M. Alonso-Gómez, Julia Wärnberg, Jesús Vioque, Dora Romaguera, José López-Miranda, Ramon Estruch, Francisco J. Tinahones, José Lapetra, Lluís Serra-Majem, Aurora Bueno-Cavanillas, Josep A. Tur, Vicente Martín-Sánchez, Xavier Pintó, José J. Gaforio, Pilar Matía-Martín, Josep Vidal, Angela Amengual-Galbarte, Lidia Daimiel, Emilio Ros, Ana García-Arellano, Rocío Barragán, Montse Fitó, Patricia J. Peña-Orihuela, Alberto Asencio-Aznar, Enrique Gómez-Gracia, Diego Martinez-Urbistondo, Marga Morey, Rosa Casas, Eva María Garrido-Garrido, Lucas Tojal-Sierra, Miguel Damas-Fuentes, Estibaliz Goñi, Carolina Ortega-Azorín, Olga Castañer, Antonio Garcia-Rios, Cristina Gisbert-Sellés, Carmen Sayón-Orea, Helmut Schröder, Jordi Salas-Salvadó, Nancy Babio

**Affiliations:** ^1^Departament de Bioquímica i Biotecnologia, Universitat Rovira i Virgili, Unitat de Nutrició Humana, Reus, Spain; ^2^Institut d'Investigació Sanitària Pere Virgili (IISPV), Reus, Spain; ^3^Consorcio CIBER (Centro de Investigación Biomédica en Red), Fisiopatología de la Obesidad y la Nutrición (CIBERObn), Instituto de Salud Carlos III, Madrid, Spain; ^4^Department of Epidemiology and Biostatistics, School of Public Health, Faculty of Medicine, Imperial College London, St Mary's Campus, London, United Kingdom; ^5^Serra Hunter Programme, Universitat Rovira i Virgili, Nutrition and Mental Health Research Group (NUTRISAM), Reus, Spain; ^6^Department of Preventive Medicine and Public Health, University of Navarra, IdiSNA (Instituto de Investigación Sanitaria de Navarra), Pamplona, Spain; ^7^Department of Preventive Medicine, University of Valencia, Valencia, Spain; ^8^Cardiovascular Risk and Nutrition Research Group (CARIN), Hospital del Mar Research Institute (IMIM), Departament de Medicina, Universitat Autònoma de Barcelona, Barcelona, Spain; ^9^Department of Nutrition, University of Navarra, Food Science and Physiology, IdiSNA (Instituto de Investigación Sanitaria de Navarra), Pamplona, Spain; ^10^Precision Nutrition Program, IMDEA (Institutos Madrileño de Estudios Avanzados) Food, CEI UAM (Comité de Ética para la Investigación-Universidad Autónoma de Madrid) + CSIC (Consejo Superior de Investigaciones Científicas), Madrid, Spain; ^11^Bioaraba Health Research Institute, Osakidetza Basque Health Service, Araba University Hospital, University of the Basque Country/Euskal Herriko Unibertsitatea UPV/EHU, Vitoria-Gasteiz, Spain; ^12^Department of Nursing, University of Málaga, Institute of Biomedical Research in Malaga (IBIMA), Málaga, Spain; ^13^Instituto de Investigación Sanitaria y Biomédica de Alicante, Miguel Hernandez University (ISABIAL-UMH), Alicante, Spain; ^14^CIBER de Epidemiología y Salud Pública (CIBERESP), Instituto de Salud Carlos III, Madrid, Spain; ^15^Health Research Institute of the Balearic Islands (IdISBa), Palma de Mallorca, Spain; ^16^Department of Internal Medicine, Maimonides Biomedical Research Institute of Cordoba (IMIBIC), Reina Sofia University Hospital, University of Cordoba, Cordoba, Spain; ^17^Department of Internal Medicine, Institutd'Investigacions Biomèdiques August Pi Sunyer (IDIBAPS), Hospital Clinic, University of Barcelona, Barcelona, Spain; ^18^Department of Endocrinology, Virgen de la Victoria Hospital, Instituto de Investigación Biomédica de Málaga (IBIMA), University of Málaga, Málaga, Spain; ^19^Department of Family Medicine, Research Unit, Distrito Sanitario Atención Primaria Sevilla, Sevilla, Spain; ^20^Preventive Medicine Service, Research Institute of Biomedical and Health Sciences (IUIBS), Centro Hospitalario Universitario Insular Materno Infantil (CHUIMI), Canarian Health Service, University of Las Palmas de Gran Canaria, Las Palmas, Spain; ^21^Department of Preventive Medicine, University of Granada, Granada, Spain; ^22^Research Group on Community Nutrition and Oxidative Stress, University of Balearic Islands, Palma de Mallorca, Spain; ^23^Institute of Biomedicine (IBIOMED), University of León, León, Spain; ^24^Lipids and Vascular Risk Unit, Internal Medicine, Hospital Universitario de Bellvitge-IDIBELL (Instituto de Investigación Biomédica de Bellvitge), Hospitalet de Llobregat, Barcelona, Spain; ^25^University of Barcelona, Barcelona, Spain; ^26^Department of Health Sciences, Instituto Universitario de Investigación en Olivar y Aceites de Oliva, University of Jaén, Jaén, Spain; ^27^Department of Endocrinology and Nutrition, Instituto de Investigación Sanitaria Hospital Clínico San Carlos (IdISSC), Universidad Complutense, Madrid, Spain; ^28^Departament of Endocrinology, Institut d'Investigacions Biomèdiques August Pi Sunyer (IDIBAPS), Hospital Clínic, University of Barcelona, Barcelona, Spain; ^29^CIBER Diabetes y Enfermedades Metabólicas (CIBERDEM), Instituto de Salud Carlos III (ISCIII), Madrid, Spain; ^30^Department of Endocrinology, Rey Juan Carlos University Hospital, Móstoles, Spain; ^31^Nutritional Control of the Epigenome Group, IMDEA (Institutos Madrileño de Estudios Avanzados) Food, CEI UAM (Comité de Ética para la Investigación-Universidad Autónoma de Madrid) + CSIC (Consejo Superior de Investigaciones Científicas), Madrid, Spain; ^32^Emergency Department, Hospital Universitario de Navarra, Servicio Navarro de Salud-Osasunbidea, Pamplona, Spain; ^33^Primary Health Care Center Muchamiel, Alicante, Spain; ^34^Department of Preventive Medicine and Public Health, School of Medicine, University of Málaga, Málaga, Spain; ^35^Institute of Biomedical Research in Malaga-IBIMA, Málaga, Spain; ^36^Department of Internal Medicine, Clinic University of Navarra, Pamplona, Spain; ^37^Primary Care Center Zaidín-Center, Servicio Andaluz de Salud, Granada, Spain; ^38^Primary Health Care Center San Vicente del Raspeig, Alicante, Spain

**Keywords:** kidney function, chronic kidney disease (CKD), glomerular filtration rate (GFR), net endogenous acid production (NEAP), potential renal acid load (PRAL), dietary acid load, albuminuria, renal nutrition

## Abstract

**Background:**

Diets high in acid load may contribute to kidney function impairment. This study aimed to investigate the association between dietary acid load and 1-year changes in glomerular filtration rate (eGFR) and urine albumin/creatinine ratio (UACR).

**Methods:**

Older adults with overweight/obesity and metabolic syndrome (mean age 65 ± 5 years, 48% women) from the PREDIMED-Plus study who had available data on eGFR (*n* = 5,874) or UACR (*n* = 3,639) at baseline and after 1 year of follow-up were included in this prospective analysis. Dietary acid load was estimated as potential renal acid load (PRAL) and net endogenous acid production (NEAP) at baseline from a food frequency questionnaire. Linear and logistic regression models were fitted to evaluate the associations between baseline tertiles of dietary acid load and kidney function outcomes. One year-changes in eGFR and UACR were set as the primary outcomes. We secondarily assessed ≥ 10% eGFR decline or ≥10% UACR increase.

**Results:**

After multiple adjustments, individuals in the highest tertile of PRAL or NEAP showed higher one-year changes in eGFR (PRAL, β: –0.64 ml/min/1.73 m^2^; 95% CI: –1.21 to –0.08 and NEAP, β: –0.56 ml/min/1.73 m^2^; 95% CI: –1.13 to 0.01) compared to those in the lowest category. No associations with changes in UACR were found. Participants with higher levels of PRAL and NEAP had significantly higher odds of developing ≥10% eGFR decline (PRAL, OR: 1.28; 95% CI: 1.07–1.54 and NEAP, OR: 1.24; 95% CI: 1.03–1.50) and ≥10 % UACR increase (PRAL, OR: 1.23; 95% CI: 1.04–1.46) compared to individuals with lower dietary acid load.

**Conclusions:**

Higher PRAL and NEAP were associated with worse kidney function after 1 year of follow-up as measured by eGFR and UACR markers in an older Spanish population with overweight/obesity and metabolic syndrome.

## Introduction

Impaired renal function is a common condition in older individuals with comorbidities as diabetes, hypertension or obesity that usually predicts the onset of Chronic Kidney Disease (CKD) (1). In the last few years, there has been a growing concern about this disease since it has a huge impact worldwide affecting around 700 million people (2). In addition, CKD is linked to several complications, such as cardiovascular events, hospitalization and/or premature death (2, 3). Consequently, appropriate and affordable prevention measures are required to preserve renal function, especially in high-risk populations (1). Prevention measures could also reduce the severe impact of CKD on the wellbeing of individuals and health systems (3–5).

Dietary habits appear to be one of the major modifiable risk factors markedly influencing renal impartment and its progression to CKD (5, 6). Additionally, the role of diet in preserving the acid-base balance of the body has recently become more relevant, given the emerging evidence linking dietary acid load with the development of different chronic diseases (7, 8), including CKD (9). It has been previously documented that healthy dietary patterns provide an alkaline environment in the body (10, 11) since plant-based food such as vegetables, fruit and some nuts or legumes have the capacity of inducing a basic environment (12). However, red and processed meats as well as ultra-processed foods are acid-producing (9, 12). Thus, these foods might be implied in the onset of a low-grade metabolic acidosis state, thereby, resulting in faster progression of kidney disease (11, 13). Overall, potential renal acid load (PRAL) and net endogenous acid production (NEAP) are the most common and suitable indexes used to estimate the acid load of the diet (9, 11). Considering the aforementioned evidence, following a healthy diet characterized by a low acid load may be a useful preventive strategy against kidney dysfunction.

To date, results from epidemiological studies focused on dietary acid load and kidney function or CKD development are inconsistent (9) and this relationship needs to be further explored. In some studies, an association between higher levels of PRAL and/or NEAP indexes and an estimated-glomerular filtration rate (eGFR) decline or higher risk of incident CKD (14–18) has been reported, but others have observed no such associations (19, 20). Also, the quality of evidence is moderate as most of the studies were mainly cross-sectional (14–17, 21, 22), and only a few were longitudinal studies (18–20). Furthermore, since most research has been conducted in healthy young or middle-aged individuals or in patients with advanced CKD, little is known about the potentially harmful association between dietary acid load and kidney function of older populations with underlying comorbid conditions. In addition, analyses assessing dietary acid load on kidney function have rarely been conducted in Mediterranean populations at high cardiovascular risk. Hence, as more scientific evidence and longitudinal studies in this field are required, we prospectively investigated the association between PRAL and NEAP and 1-year changes in two markers of kidney function decline, eGFR and Urine Albumin/Creatinine Ratio (UACR), in a large Spanish cohort of older adults with overweight/obesity and metabolic syndrome (MetS).

## Materials and methods

### Study population and design

The present study is a prospective analysis of baseline and 1-year data within the framework of the PREvención con DIeta MEDiterránea (PREDIMED)-Plus trial. Briefly, the PREDIMED-Plus is an ongoing, parallel-group, randomized and controlled clinical trial aiming to assess the effect of an intensive weight loss intervention on cardiovascular disease (CVD) morbidity and mortality. An energy-restricted Mediterranean diet (MedDiet), physical activity promotion and behavioral support are compared to usual care advice in 6,874 older adults enrolled between 2013 and 2016 by 23 Spanish recruitment centers. Eligible participants were men aged 55–75 years and women aged 60–75 years with overweight or obesity [Body Mass Index (BMI) 27–40 kg/m^2^], who satisfied at least 3 criteria for the MetS (23). Further details of the inclusion and exclusion criteria and the study design have been described elsewhere (24). A detailed explanation of the protocol is also available at https://www.predimedplus.com. This trial was registered on the International Standard Randomized Controlled Trial registry (https://www.isrctn.com/ISRCTN89898870) with number 89898870 in July of 2014. The final study protocol and procedures were approved following the standards of the Declaration of Helsinki by the Institutional Review Boards of participating centers and all participants provided written informed consent.

For the current study, participants without completed food frequency questionnaire (FFQ) information and reporting implausible total energy intake (men < 800 and >4,000 kcal/day and women < 500 and >3,500 kcal/day) at baseline were excluded (*n* = 227) from the analyses (25). We also excluded participants who died (*n* = 11) or were lost to follow-up (*n* = 16) during the first year. Moreover, participants with missing data on eGFR (*n* = 746) or UACR (*n* = 2,981) at baseline and/or at the 1-year assessment were excluded when eGFR or UACR were the outcomes, respectively. Therefore, a final sample of 5,874 participants for eGFR and 3,639 participants for UACR were analyzed (Supplementary Figure 1).

### Assessment of dietary intake and dietary acid load

To evaluate dietary intake, trained dieticians administered a 143-item FFQ, based on a previously validated one for the Spanish population (26), in face-to-face interviews at baseline. Each participant was asked about their frequency of consumption during the preceding year of each specific item, which had nine possible answers ranging from never to more than 6 times per day. The typical portion size of each item was subsequently transformed into grams or milliliters per day, as appropriate. Two Spanish food composition databases were referenced to calculate total daily energy and nutrient intake (27, 28).

Dietary acid load was estimated at baseline using individual nutritional data obtained from the FFQ. Previously published methods proposed by Remer and Manz (29) and Frassetto et al. (8) were applied for the calculation of PRAL and NEAP scores, respectively. PRAL (mEq/day) = 0.4888 × protein intake (g/day) + 0.0366 × phosphorus (mg/day) – 0.0205 × potassium (mg/day) – 0.0125 × calcium (mg/day) – 0.0263 × magnesium (mg/day). NEAP (mEq/day) = 54.5 × protein (g/day)/potassium (mEq/day) – 10.2.

### Ascertainment of the outcome

Serum creatinine (SCr) levels and urinary creatinine and albumin concentrations were determined using routine laboratory methods from blood and spot morning urine samples collected at baseline and 1-year following overnight fasting. For the current study, 1-year changes in eGFR and UACR were considered our primary outcomes. We indirectly determined eGFR from SCr using the Chronic Kidney Disease Epidemiology Collaboration equation for Caucasian individuals (CKD-EPI) (30) and the UACR was calculated by dividing urine albumin (mg/l) by urine creatinine concentrations (mg/l). UACR values were truncated at 500 mg/g to minimize the influence of outliers. There were 21 observations > 500 mg/g at baseline and 24 at 1 year that were >500 mg/g and subsequently set to 500 mg/g. One-year changes in both eGFR and UACR were calculated by subtracting values at 1 year minus values at baseline. Secondary outcomes were ≥10% eGFR decline and ≥10% UACR increase following a 1-year follow-up. These were estimated by applying the formula: [(1-year eGFR or UACR – baseline eGFR or UACR)/baseline eGFR or UACR]^*^100. Participants were categorized as those with a ≥10% or <10% eGFR decline (31) or with a ≥10% or <10% increase in UACR.

### Covariate assessment

At baseline, trained PREDIMED-Plus staff collected socio-demographic and lifestyle information including age, sex, educational level, physical activity, smoking status, as well as medication use and history of disease using several questionnaires or reviewing medical records. Moreover, adherence to the energy-reduced MedDiet was evaluated using a validated 17-item MedDiet questionnaire (32). Compliance with each item of the MedDiet questionnaire was scored with one point and non-compliance with 0. Thereafter, a cut-off point based on the median of the score was determined by dividing individuals into those with high adherence to a MedDiet (≥9 points) or a low adherence (<9 points). Moreover, other cut-off points were tested arbitrarily and defined as the highest tertiles or quartiles (in both cases high adherence was observed to be ≥12 points). Total daily energy intake and sodium intake were estimated according to data from the FFQ. Anthropometric variables were measured in duplicate and resting blood pressure was measured in triplicate using an automated digital device (Omron-HEM297705C). BMI was calculated as weight in kilograms divided by the square of height in meters. In our analysis, white blood cell count was used to assess inflammation (leucocytes > 10 × 10^9^/L).

### Statistical analyses

For the present report, we used the PREDIMED-Plus database generated in December 2020. Participants were categorized into tertiles of PRAL and NEAP. One-way ANOVA and chi-square tests were used to evaluate differences among tertiles of PRAL and NEAP for the baseline characteristics of the study population. Descriptive data were expressed as means ± SD for continuous variables and percentages (%) and numbers for categorical variables.

Multivariate linear regression models were performed to examine the associations between tertiles of PRAL and NEAP at baseline and 1-year changes in eGFR (ml/min/1.73 m^2^) and UACR (mg/g). For these associations, PRAL and NEAP were also analyzed as continuous variables (both for each 1-SD increase). β-coefficients and 95% confidence intervals (CIs) were assessed using two different models: Model 1 was adjusted for sex and age; and Model 2 was further adjusted for study center (categorized into quartiles by number of participants), intervention group (treatment/control), BMI (kg/m^2^), smoking status (never/current/former smoker), educational level (primary education/secondary education/graduate), leisure-time physical activity (METs/min/week, tertiles), diabetes prevalence (yes/no), hypertension prevalence (yes/no), hypercholesterolemia prevalence (yes/no), angiotensin-converting enzyme inhibitors (ACEis) (yes/no), angiotensin II receptor blockers (ARBs) (yes/no), MedDiet adherence (high/low adherence), energy intake (kcal/day, tertiles), sodium intake (mg/day, tertiles) and high leukocyte levels (yes/no). Moreover, odds ratios (OR) and their 95% CIs were calculated for the association between tertiles of NEAP and PRAL and ≥10% eGFR decline and ≥10% UACR increase at 1-year of follow-up adjusting for the same confounders as mentioned in model 2. The first tertile was used as a reference category in all regression models. Additionally, linear regression models were further adjusted for baseline eGFR (ml/min/1.73 m^2^) or baseline UACR (mg/g) depending on the main outcome. Variance inflation factors (VIFs) were used to assess collinearity for the multivariable models and, as VIFs were <2.5, none of the covariates needed to be removed. All analyses were conducted with robust estimates of the variance to correct for possible intra-cluster correlation. Intra-cluster was defined as the participants who shared the same household. To assess the linear trend, the median value of each tertile of PRAL and NEAP were modeled as continuous variables.

We also conducted subgroup analyses for the 1-year changes in eGFR and UACR stratifying by baseline categories of eGFR (≥90; 60–90; <60 ml/min/1.73 m^2^) and UACR (<30; ≥30 mg/g). Interaction between tertiles of PRAL and NEAP with categories of eGFR, UACR, and energy-reduced MedDiet adherence (high/low), as well as the intervention/control group were checked in the fullest multivariable model using likelihood ratio tests and non-significant results were observed. In a sensitivity analysis, we repeated our main analysis investigating the association between PRAL and NEAP with 1-year changes in eGFR and UACR after excluding individuals with eGFR < 60 ml/min/1.73 m^2^ or with UACR > 300 mg/g at baseline. In addition, as a supplementary analysis, we evaluated the association between dietary acid load and ≥5% eGFR decline and ≥5% UACR increase following the same procedure mentioned previously. Statistical analyses were conducted using Stata/SE software, version 14.0 (StataCorp, College Station, TX) and significance level was set at a 2-tailed *p* < 0.05.

## Results

Table 1 shows the baseline characteristics of the study population according to tertiles of PRAL and NEAP. In general, participants with higher values of PRAL and NEAP at baseline were more likely to be younger, men, have a higher BMI, smoke, have a higher educational level, and were less likely to exercise. Participants in the highest tertiles of PRAL and NEAP also had higher levels of creatinine and eGFR than those in the lowest tertile. In terms of mediations, participants in the highest tertiles of PRAL and NEAP were more likely to have used insulin, ACEis treatment, and took less antihypertensive and ARB drugs. Furthermore, individuals in the highest tertile of NEAP were more likely to have type 2 diabetes. However, no significant differences were observed between tertiles of PRAL nor NEAP regarding the UACR or CKD. Concerning dietary assessment, adherence to an energy-reduced MedDiet was lower in individuals with higher dietary acid load levels than those in the lowest tertile of PRAL and NEAP. Moreover, participants in the highest tertile of PRAL and NEAP had a lower intake of vegetable/animal protein ratio, carbohydrates and fiber while they had a higher energy, protein and fat consumption than those with low values of both dietary acid load indexes. Similar trends were observed when baseline consumption of food groups across tertiles of PRAL and NEAP were analyzed (Supplementary Table 1). Supplementary Table 2 presents further information regarding macronutrient and micronutrient intake, especially those related to dietary acid load, at 1-year of follow-up. Baseline characteristics according to included and excluded participants from the eGFR or UACR analyses are described in Supplementary Table 3.

**Table 1:** Baseline characteristics of the study population with data on eGFR at 1-year follow-up by tertiles of PRAL and NEAP (*n* = 5,874).

		**PRAL (mEq/d)**				**NEAP (mEq/d)**			
	**Total**	**T1**	**T2**	**T3**		**T1**	**T2**	**T3**	
	***n* = 5,874**	***n* = 1,958**	***n* = 1,958**	***n* = 1,958**	***p*-value**	***n* = 1,958**	***n* = 1,958**	***n* = 1,958**	***p*-value**
PRAL, mEq/day	−5.4 ± 15.6	–	–	–	–	−21.4 ± 11.3	−5.0 ± 5.4	10.1 ± 8.7	< 0.01
NEAP, mEq/day	36.9 ± 8.1	29.0 ± 3.8	36.4 ± 2.8	45.6 ± 6.0	<0.01	–	–	–	–
Age, years	65.0 ± 4.9	65.7 ± 4.7	65.1 ± 5.0	64.2 ± 4.9	<0.01	65.7 ± 4.7	65.3 ± 4.9	64.1 ± 4.9	<0.01
Women, % (*n*)	48.0 (2,818)	52.7 (1,31)	49.1 (961)	42.2 (826)	<0.01	56.0 (1,097)	48.9 (957)	39.0 (764)	<0.01
Intervention group, % (*n*)	49.4 (2,901)	49.3 (966)	50.3 (984)	48.6 (951)	0.57	50.0 (978)	49.9 (976)	48.4 (947)	0.54
BMI, kg/m^2^	32.5 ± 3.4	32.4 ± 3.4	32.4 ± 3.4	32.8 ± 3.5	<0.01	32.3 ± 3.4	32.5 ± 3.4	32.7 ± 3.5	<0.01
PA, METS/min/week	2,528.0 ± 2,350.4	2,740.2 ± 2,483.6	2,526.2 ± 2,342.2	2,317.7 ± 2,198.8	<0.01	2,681.7 ± 2,434.1	2,547.4 ± 2,373.8	2,355.1 ± 2,228.2	<0.01
Smoking status, % (*n*)					<0.01				<0.01
Never smoked	44.4 (2,605)	47.9 (939)	45.5 (891)	39.6 (775)		49.9 (976)	44.7 (875)	38.5 (754)	
Former smoker	43.0 (2,528)	40.3 (789)	42.3 (828)	46.5 (911)		38.6 (756)	43.0 (842)	47.5 (930)	
Current smoker	12.6 (741)	11.8 (230)	12.2 (239)	13.9 (272)		11.5 (226)	12.3 (241)	14.0 (274)	
Education level, % (*n*)					<0.01				<0.01
Primary education	49.22 (2,891)	54.9 (1,075)	49.2 (963)	43.6 (853)		54.0 (1,058)	50.0 (979)	44.6 (854)	
Secondary education	29.18 (1,714)	25.2 (494)	28.9 (565)	33.4 (655)		25.6 (501)	28.3 (555)	33.6 (658)	
College/university	21.60 (1,269)	19.9 (374)	22.0 (430)	23.0 (450)		20.4 (399)	21.7 (424)	22.8 (446)	
Creatinine	0.8 ± 0.2	0.8 ± 0.2	0.8 ± 0.2	0.9 ± 0.2	<0.01	0.8 ± 0.2	0.8 ± 0.2	0.9 ± 0.2	<0.01
eGFR, ml/min/1.73 m^2^	84.2 ± 13.9	83.6 ± 13.6	84.7 ± 13.9	84.3 ± 14.4	0.04	83.5 ± 13.6	84.9 ± 13.7	84.1 ± 14.5	<0.01
UACR, mg/g	16.8 ± 48.9	16.2 ± 45.0	16.8 ± 50.0	17.5 ± 51.5	0.78	16.4 ± 46.4	15.9 ± 47.0	18.2 ± 53.1	0.42
CKD, % (*n*)	4.4 ± 3.7	6.5 (126)	6.5 (128)	6.8 (133)	0.79	6.6 (129)	5.8 (113)	7.6 (145)	0.10
Type 2 diabetes, % (*n*)	30.6 (1,797)	28.9 (567)	30.7 (601)	32.1 (629)	0.10	28.8 (564)	30.4 (595)	32.6 (638)	0.04
Hypertension, % (*n*)	84.1 (4,941)	85.1 (1,666)	84.5 (1,654)	82.8 (1,621)	0.13	85.1 (1,667)	84.4 (1,653)	82.8 (1,621)	0.12
Hypercholesterolemia, % (*n*)	69.7 (4,096)	69.4 (1,359)	69.2 (1,356)	70.5 (1,381)	0.63	70.2 (1,375)	69.3 (1,356)	69.7 (1,365)	0.80
Hypertriglyceridemia, % (*n*)^*^	39.7 (2,327)	40.7 (795)	39.1 (763)	42.5 (831)	0.10	40.8 (795)	39.2 (765)	42.4 (829)	0.13
Low HDL, % (*n*)^†^	40.8 (2,389)	38.5 (751)	39.4 (768)	41.3 (808)	0.18	39.1 (763)	39.0 (760)	41.1 (804)	0.30
**Medication use, % (** ***n*** **)**
Lipid-lowering drugs	51.8 (3, 42)	52.6 (1, 30)	51.7 (1, 13)	51.0 (999)	0.66	52.8 (1, 35)	51.9 (1, 17)	50.6 (990)	0.35
Oral blood glucose-lowering drugs	26.0 (1,528)	25.2 (494)	26.3 (516)	26.5 (518)	0.62	25.3 (495)	25.7 (504)	27.0 (529)	0.44
Insulin treatment	4.1 (239)	3.5 (68)	3.7 (73)	5.0 (98)	0.03	3.2 (64)	4.1 (81)	4.8 (94)	0.05
Antihypertensive drugs	78.7 (4,625)	81.7 (1,599)	77.9 (1,525)	76.7 (1,501)	<0.01	81.0 (1,585)	79.1 (1,549)	76.2 (1,491)	<0.01
ARBs	36.3 (2,131)	39.6 (776)	34.9 (683)	34.3 (672)	<0.01	39.5 (774)	35.2 (689)	34.1 (668)	<0.01
ACEis	30.2 (1,775)	28.6 (559)	31.2 (611)	30.9 (605)	0.11	27.9 (546)	32.0 (624)	30.9 (605)	0.02
**Dietary assessment**
erMedDiet score, 17-points	8.5 ± 2.7	9.2 ± 2.6	8.4 ± 2.7	7.9 ± 2.5	<0.01	9.3 ± 2.6	8.6 ± 2.6	7.7 ± 2.5	<0.01
Energy intake, kcal/d	2,370.5 ± 548.9	2,366.1 ± 537.9	2,303.1 ± 531.8	2,442.3 ± 567.8	<0.01	2,277.5 ± 531.2	2,278.6 ± 533.1	2,455.7 ± 562.5	<0.01
Protein intake, % energy	16.7 ± 2.8	16.1 ± 2.6	16.7 ± 2.7	17.4 ± 3.0	<0.01	16.1 ± 2.7	16.9 ± 2.7	17.3 ± 2.9	<0.01
Vegetal /animal protein ratio, g/d	0.5 ± 0.2	0.67 ± 0.27	0.56 ± 0.19	0.48 ± 0.17	<0.01	0.68 ± 0.28	0.56 ± 0.19	0.49 ± 0.17	<0.01
Fat intake, % energy	39.6 ± 6.5	38.4 ± 6.4	39.7 ± 6.4	40.8 ± 6.5	<0.01	38.5 ± 6.5	39.5 ± 6.3	40.9 ± 6.5	<0.01
Carbohydrate intake, % energy	40.5 ± 6.8	42.4 ± 6.6	40.4 ± 6.5	38.7 ± 6.8	<0.01	42.4 ± 6.8	40.5 ± 6.4	38.6 ± 6.8	<0.01
Fiber intake, g/day	26.1 ± 8.7	30.4 ± 9.1	25.2 ± 7.8	22.8 ± 7.5	<0.01	29.9 ± 9.5	26.4 ± 7.9	22.2 ± 7.0	<0.01
Potassium intake, mg/day	4,477.0 ± 1,079.6	5,108.6 ± 1,124.3	4,313.1 ± 898.2	4,009.2 ± 884.4	<0.01	4,953.4 ± 1,189.3	4,501.7 ± 924.5	3,975.8 ± 866.0	<0.01
Calcium intake, mg/day	1,034.0 ± 347.0	1,062.8 ± 353.6	999.2 ± 327.9	1,040.1 ± 355.9	<0.01	1,030.0 ± 350.5	1,049.6 ± 337.1	1,022.5 ± 352.7	0.04
Magnesium intake, mg/day	420.4 ± 108.2	457.7 ± 112.5	407.6 ± 102.2	396.0 ± 99.3	<0.01	446.2 ± 117.8	425.2 ± 102.5	389.8 ± 95.6	<0.01
Phosphorus intake, mg/day	1,759.1 ± 419.9	1,750.1 ± 429.1	1,703.8 ± 401.9	1,823.5 ± 419.7	<0.01	1,713.1 ± 438.3	1,783.5 ± 403.4	1,780.8 ± 413.6	<0.01
Sodium intake, mg/day	2,430.0 ± 774.8	2,272.5 ± 736.8	23,183.0 ± 679.8	2,699.4 ± 828.6	<0.01	2,187.8 ± 712.4	2,412.4 ± 689.3	2,689.6 ± 832.1	<0.01

ACEis, Angiotensin-Converting Enzyme Inhibitors; ARBs, Angiotensin II receptor blockers; eGFR, Estimated Glomerular Filtration Rate; erMedDiet, energy-restricted Mediterranean diet; HDL, High-Density Lipoprotein; NEAP, Net Endogenous Acid Production; MET, Metabolic Equivalent of Task T, tertile; BMI, Body Mass Index; PRAL, Potential Renal Acid Load; PA, Physical activity; eGFR, estimated Glomerular Filtration Rate; CKD, Chronic Kidney Disease (eGFR < 60 ml/min/1.73 m^2^); UACR, Urine Albumin/Creatinine Ratio. Values are presented as percentages (n) for categorical variables and means ± standard deviations for continuous variables. P-value was calculated by chi-square or one-way analysis of variance test for categorical and continuous variables, respectively.

* Fasting triglyceride concentration ≥150 mg/dL or specific treatment for lipid abnormality.

† HDL concentration <40 mg/dL in men and <50 mg/dL in women or specific treatment for lipid abnormality.

The association (β-coefficient; 95% CI) between tertiles of PRAL and NEAP and 1-year changes in eGFR and UACR are displayed in Table 2. In the most adjusted model, PRAL showed a significant inverse association with 1-year changes in eGFR (β: −0.17 ml/min/1.73 m^2^; 95% CI: −0.71 to 0.36 for T2 vs. T1, β: −0.64 ml/min/1.73 m^2^; 95% CI: −1.21 to −0.08 for T3 vs. T1). We found similar results when PRAL and NEAP were analyzed as continuous variables (PRAL: β: −0.25 ml/min/1.73 m^2^; 95% CI: −0.47 to −0.03 for each 1-SD increment. NEAP: β: −0.28 ml/min/1.73 m^2^; 95% CI: −0.51 to −0.05 for each 1-SD increment). Results remained essentially the same after adding 1-year BMI change to the most adjusted model (data not shown). PRAL and NEAP were not significantly associated with UACR changes after 1-year of follow-up after modeling them as tertiles, nor as continuous variables. In the sensitivity analyses, excluding individuals with <60 ml/min/1.73 m^2^ of eGFR or with >300 mg/g of UACR did not modify the main findings for both outcomes (data not shown). When we repeated the principal analyses, stratifying by baseline categories of eGFR (≥90; 60–90; < 60 ml/min/1.73 m^2^) and UACR (<30; ≥30 mg/g), the results presented a similar tendency (Supplementary Table 4). In participants with eGFR ≥ 90 ml/min/1.73 m^2^, significant associations were observed with eGFR changes when both dietary acid load indexes were modeled as continuous variables (PRAL: β: −0.28 ml/min/1.73 m^2^; 95% CI: −0.56 to −0.01 for each 1-SD increment. NEAP: β: −0.31 ml/min/1.73 m^2^; 95% CI: −0.58 to −0.03 for each 1-SD increment). The main analysis was repeated using other cut-offs points for the MedDiet score confounding factor (i.e., ≥12 points for high adherence) and similar results were found (Supplementary Table 5). We also explored the interactions between tertiles of PRAL and NEAP and the adherence to energy-reduced MedDiet, categories of eGFR and UACR, as well as intervention/control group, and no statistically significant findings were observed (all interactions, *p* > 0.05).

**Table 2:** Multivariable-adjusted β-coefficients and 95% CI of 1-year changes in eGFR (ml/min/1.73 m^2^) or in UACR (mg/g) across tertiles and per 1-SD increment of baseline PRAL and NEAP.

	**PRAL (mEq/d)**						
	**T1**	**T2**	**T3**	***p*** **for trend**	Continuous (1 SD^**^)
	***n*** **=** **1,958**	***n*** **=** **1,958**	***n*** **=** **1,958**		***n*** **=** **5,874**
**Δ** **in eGFR, ml/min/1.73 m**^**2**^	−0.69 (−1.07 to −0.31)	−0.86 (−1.24 to −0.49)	−1.34 (−1.72 to −0.95)		
Model 1	0 (Ref.)	−0.16 (−0.70 to 0.37)	−0.52 (−1.06 to 0.03)	0.062	−0.21 (−0.42 to 0.01)
Model 2	0 (Ref.)	−0.17 (−0.71 to 0.36)	−0.64 (−1.21 to −0.08)^*^	0.026	−0.25 (−0.47 to −0.03)^*^
	***n*** **=** **1,213**	***n*** **=** **1,213**	***n*** **=** **1,213**		***n*** **=** **3,639**
**Δ** **in UACR, mg/g**	4.37 (1.96 to 6.78)	2.74 (0.60 to 4.88)	1.39 (−0.62 to 3.39)		
Model 1	0 (Ref.)	−1.20 (−4.32 to 1.93)	−2.31 (−5.28 to 0.66)	0.128	−0.88 (−2.00 to 0.25)
Model 2	0 (Ref.)	−1.63 (−4.84 to 1.58)	−2.99 (−6.34 to 0.37)	0.082	−1.22 (−2.51 to 0.08)
**NEAP (mEq/d)**					
	***n*** **=** **1,958**	***n*** **=** **1,958**	***n*** **=** **1,958**		***n*** **=** **5,874**
**Δ** **in eGFR, ml/min/1.73 m**^**2**^	−0.68 (−1.06 to −0.30)	−0.97 (−1.35 to −0.60)	−1.24 (−1.63 to −0.84)		
Model 1	0 (Ref.)	−0.28 (−0.81 to 0.25)	−0.44 (−0.99 to 0.11)	0.116	−0.22 (−0.44 to −0.01)^*^
Model 2	0 (Ref.)	−0.30 (−0.83 to 0.24)	−0.56 (−1.13 to 0.01)	0.056	−0.28 (−0.51 to −0.05)^*^
	***n*** **=** **1,213**	***n*** **=** **1,213**	***n*** **=** **1,213**		***n*** **=** **3,639**
**Δ** **in UACR, mg/g**	3.92 (1.49 to 6.34)	3.09 (1.14 to 5.03)	1.49 (−0.54 to 3.53)		
Model 1	0 (Ref.)	−0.81 (−3.82 to 2.20)	−1.96 (−5.07 to 1.15)	0.214	−0.93 (−2.13 to 0.28)
Model 2	0 (Ref.)	−0.83 (−3.87 to 2.21)	−2.42 (−5.79 to 0.95)	0.154	−1.26 (−2.63 to 0.10)

*eGFR*, Estimated glomerular filtration rate; *NEAP*, Net Endogenous Acid Production; *T*, tertile; *PRAL*, Potential Renal Acid Load; *UACR*, Urine albumin/creatinine ratio. Model 1: adjusted for age (years), sex and baseline eGFR or baseline UACR (in continuous, depending on the main outcome). Model 2: additionally adjusted for participating center (categorized into quartiles by number of participants), intervention group (treatment/control), body mass index (kg/m^2^), smoking habits (never, current or former smoker), educational level (primary, secondary education or graduate), leisure-time physical activity (METS/min/week in tertiles), diabetes prevalence (yes/no), hypertension prevalence (yes/no) and hypercholesterolemia prevalence (yes/no), ARBs (yes/no), ACEis (yes/no), Mediterranean diet adherence (high/low adherence), energy intake (kcal/day in tertiles), sodium intake (mg/g in tertiles) and high leukocytes levels (yes/no).

* *p*-value < 0.05.

** One SD = 15.6 mEq/d in PRAL and 8.1 mEq/d in NEAP.

**Figure 1:**
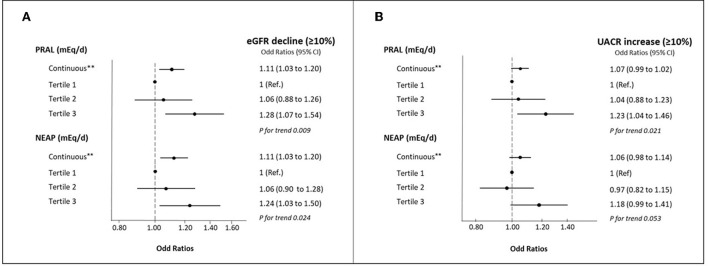
**(A)** Multivariable-adjusted OR (95% CIs) for ≥10% eGFR decline by tertiles of baseline PRAL and NEAP and per 1-SD increment. **(B)** Multivariable-adjusted OR (95% CIs) for ≥10% UACR increase by tertiles of baseline PRAL and NEAP and per 1-SD increment. eGFR, Estimated glomerular filtration rate; NEAP, Net Endogenous Acid Production; T, tertile; PRAL, Potential Renal Acid Load; UACR, Urine albumin/creatinine ratio. Percentage of participants with eGFR decline (>10%): tertile 1 of PRAL (*n* = 296; % = 15.1), tertile 2 of PRAL (*n* = 304; % = 15.5), tertile 3 of PRAL (*n* = 346; % = 17.7); tertile 1 of NEAP (*n* = 297; % = 15.2), tertile 2 of NEAP (*n* = 312; % = 15.9). tertile 3 of NEAP (*n* = 337; % = 17.2). Percentage of participants with UACR increase (>10%): tertile 1 of PRAL (*n* = 539; % = 44.4), tertile 2 of PRAL (*n* = 547; % = 45.1), tertile 3 of PRAL (*n* = 597; % = 49.2); tertile 1 of NEAP (*n* = 550; % = 45.3), tertile 2 of NEAP (*n* = 539; % = 44.4), tertile 3 of NEAP (*n* = 594; % = 49.0). All models were adjusted for age (years), sex, participating center (categorized into quartiles by number of participants), intervention group (treatment/control), body mass index (kg/m^2^), smoking habits (never, current or former smoker), educational level (primary, secondary education or graduate), leisure-time physical activity (METS/min/week in tertiles), diabetes prevalence (yes/no), hypertension prevalence (yes/no) and hypercholesterolemia prevalence (yes/no), ARBs (yes/no), ACEis (yes/no), Mediterranean diet adherence (high/low adherence), energy intake (kcal/day in tertiles), sodium intake (mg/g in tertiles), and high leukocytes levels (yes/no). **one SD = 15.6 mEq/d in PRAL and 8.1 mEq/d in NEAP.

Figure 1 depicts the OR and 95% CI for ≥10% eGFR decline and ≥10% UACR increase according to tertiles of PRAL and NEAP. After multiple adjustments, participants in the highest tertile of PRAL and NEAP were significantly more likely to have a ≥10% eGFR decline after 1 year of follow up compared to those in the lowest tertile, with ORs of 1.28 (95% CI: 1.07–1.54) for PRAL and 1.24 (95% CI: 1.03–1.0) for NEAP. When PRAL and NEAP were modeled as continuous variables (per each 1-SD increment) higher ORs were also observed. Compared to participants with low PRAL values at baseline, participants with the highest levels had a 23% (95% CI: 1.04–1.46) higher odds of ≥10% UACR increase after 1 year of follow-up after adjusting for potential confounders. No significant associations were found between NEAP and the odds of ≥10% UACR increase or for 1-SD increment of PRAL and NEAP. When a ≥5% eGFR decline and a ≥5% UACR increase were assessed, the same results were found (Supplementary Table 6).

## Discussion

The results of this prospective study conducted in older Spanish adults at high cardiovascular disease risk suggest that PRAL and NEAP are inversely associated with 1-year changes in eGFR, but not with 1-year UACR changes. Furthermore, participants with higher levels of both estimates of dietary acid load had higher odds of a ≥10% eGFR decline, and those in the highest tertile of PRAL had 23% higher odds of a ≥10% UACR increase. GFR and albuminuria are the main complementary biomarkers used in epidemiological studies to assess kidney function (3). As far as we know, this is the first study to prospectively evaluate the association between dietary acid load and kidney function concurrently assessing eGFR and UACR in a population of older adults with underlying comorbidities.

A large body of evidence has linked dietary acid load with kidney outcomes in several studies (9). However, to the best of our knowledge, there are only four cross-sectional studies and one longitudinal study investigating the potential relationship of dietary acid load with renal function defined by eGFR and/or CKD in older adults without CKD. These cross-sectional studies conducted in different cohorts of adults reported that higher dietary acid load was associated with higher odds of CKD and/or impaired kidney function as indicated by low eGFR after adjusting for multiple confounders (14, 16, 17, 33). Our observations are in accordance with these cross-sectional studies since we observed a greater eGFR decline at 1 year with higher PRAL and NEAP scores, even after adjusting for baseline eGFR and other essential confounding factors. Interestingly, our supplementary stratified analyses according to categories of eGFR, which have seldom been performed in previous studies, revealed a similar non-significant tendency to worsen kidney function with increased dietary acid load. Consistent with our findings, the prospective analysis from the cohort of the Atherosclerosis Risk in Communities (ARIC) study of 15,055 apparently healthy middle-aged participants with preserved kidney function showed that higher levels of PRAL were associated with a 13% higher risk of CKD incidence over 21 years of follow-up (18).

Regarding albuminuria, which is considered a reliable marker of kidney damage (3), preceding studies have assessed its cross-sectional association with dietary acid load obtaining inconclusive findings. In The Jackson Heart Study, there was no association between estimated Net Acid Excretion (NAE_es_) and albuminuria (16). In contrast, the NHANES study reported a positive association between dietary acid load and albuminuria in 12,293 healthy American adults (17). Additionally, the researchers from The Uonuma CKD Cohort Study also found that higher NEAP was associated with a higher UACR and risk of albuminuria among 6,684 middle-aged Japanese adults (21). To date, no large prospective cohort study has focused on the relationship between dietary acid load and albuminuria in vulnerable older adults. In the current study, we report no association between PRAL and NEAP scores and 1-year changes in UACR. This could suggest that high dietary acid load may promote tubule-interstitial injury rather than glomerular damage. Nevertheless, we were not able to check this tubular damage hypothesis since spot/24 h total proteinuria data were not available in our dataset (34). However, it is worthwhile to mention that when UACR was also assessed as an increase ≥10% after 1 year of follow-up, which is a more clinical approach, we found a significant association with PRAL. Consequently, future longitudinal studies and clinical trials would be helpful to clarify these observations related to albuminuria and dietary acid load.

Overall, our findings in conjunction with the evidence available to date, suggests that following a diet with a low acid load could be an appropriate measure to improve renal function and, accordingly, decrease the risk of CKD development and progression among older individuals from middle-aged to elderly with underlying comorbid conditions.

The potential mechanisms by which high dietary acid load may induce kidney dysfunction are unclear, though possible mechanisms have been proposed for consideration. Acid retention has been proposed to activate the intracellular renin-angiotensin system, through the previous stimulation of aldosterone production, which might be implicated in the onset or progression of kidney damage (35, 36). Moreover, metabolic acidosis appears to contribute to endothelin-1 production, which in turn could be related to tubulointerstitial injury (37–39). Besides, high dietary acid load would also induce tubular toxicity activating the complement pathway and increasing renal medullary ammonia concentrations (40–42). There is also a high probability that acid retention increases the production of oxygen-free radicals and oxidative stress (43, 44). Consequently, it is crucial for kidney health to maintain appropriate levels of acid load, and diet may play an important role in this respect (11). It should be noted that in our study individuals with high levels of dietary acid load reported higher intakes of some food groups which have been directly or indirectly implicated in kidney function damage, such as total and animal protein intake (33, 45) or sugar and sweetened products (46). By contrast, as dietary acid load increased there was a lower consumption of fiber-rich foods, including fruits, vegetables, whole-grain cereals, and nuts. Thus, the potential beneficial effects of fiber on the kidney (47) could be lacking in those individuals with high dietary acid load.

This study has some limitations that deserve to be mentioned. First, the population consisted of older Spanish individuals at high cardiovascular risk, meaning the findings may not be generalizable to other populations. Furthermore, the Mediterranean lifestyle could imply healthier habits which, at the same time, may result in different macro- and micronutrients intake related to kidney function, such as potassium-rich or low-sodium dietary intakes. Second, as PREDIMED-Plus is a randomized controlled trial, though, all the analyses were adjusted for the intervention group, the lifestyle advice that participants received could be affecting our findings. Third, dietary acid load was calculated using PRAL and NEAP from dietary nutrient intake information obtained from FFQ data. Although this questionnaire was validated and carefully administered by trained dietitians, potential measurement errors and reporting bias could be present. Fourth, while SCr-based eGFR was used as a biomarker of kidney function, as is common in most epidemiologic studies, there are other more optimal markers such as inulin, iothalamate or 24-h urinary creatinine clearance. Nevertheless, these procedures are expensive, time-consuming, and difficult measure in large populations. Finally, as in any observational study, although a substantial number of confounding factors were considered, confounding bias could not be completely ruled out and direct causality cannot be inferred. However, our study also has several strengths. Analyses were conducted using data from a large cohort, which has a wide selection of different variables to adjust the models for kidney function related-potential confounders. Moreover, it is important to highlight the prospective design that we performed and the joint assessment of two commonly used biomarkers of renal function. Another novel aspect of this study is the sensitivity and supplementary analyses conducted which gave robustness to the main results.

## Conclusion

In conclusion, the current study conducted in a population of older Spanish adults with overweight/obesity and MetS shows that higher dietary acid load is associated with changes toward a worse eGFR and higher odds of ≥10% eGFR decline and ≥10% UACR increase. Nevertheless, further longitudinal and interventional studies are needed to clarify and confirm the consistency of these associations before considering a reduction in dietary acid load as part of strategies for preventing kidney function decline.

## Data availability statement

There are restrictions on the availability of data for the PREDIMED-Plus trial, due to the signed consent agreements around data sharing, which only allow access to external researchers for studies following the project purposes. Requestors wishing to access the PREDIMED-Plus trial data used in this study can make a request to the PREDIMED-Plus trial Steering Committee chair: predimed_plus_scommitte@googlegroups.com. The request will then be passed to members of the PREDIMED-Plus Steering Committee for deliberation.

## Ethics statement

The studies involving human participants were reviewed and approved by the ethical standards of the Declaration of Helsinki by the Institutional Review Boards (IRBs). The patients/participants provided their written informed consent to participate in this study.

## Author contributions

CV-H, NB-T, AD-L, ZV-R, IM, DC, AG, JM, ÁA-Gó, JW, JVio, DR, JL-M, RE, FT, JL, LS-M, AB-C, JT, VM-S, XP, JG, PM-M, JVid, AA-Ga, LD, ER, AG-A, RB, MF, PP-O, AA-A, EG-G, DM-U, MM, RC, EMG-G, LT-S, MD-F, EG, CO-A, OC, AG-R, CG-S, CS-O, HS, JS-S, and NB designed and conducted the research. CV-H and AD-L analyzed the data. CV-H, NB-T, AD-L, and NB wrote the article. CV-H and AD-L are the guarantors of this work and, as such, had full access to all the data in the study and take responsibility for the integrity of the data and the accuracy of the data analysis. All authors revised the manuscript for important intellectual content and read and approved the final manuscript.

## Funding

This work was supported by the official Spanish Institutions for funding scientific biomedical research, CIBER Fisiopatología de la Obesidad y Nutrición (CIBEROBN) and Instituto de Salud Carlos III (ISCIII), through the Fondo de Investigación para la Salud (FIS), which is co-funded by the European Regional Development Fund (six coordinated FIS projects leaded by JS-S and JVid, including the following projects: PI13/00673, PI13/00492, PI13/00272, PI13/01123, PI13/00462, PI13/00233, PI13/02184, PI13/00728, PI13/01090, PI13/01056, PI14/01722, PI14/00636, PI14/00618, PI14/00696, PI14/01206, PI14/01919, PI14/00853, PI14/01374, PI14/00972, PI14/00728, PI14/01471, PI16/00473, PI16/00662, PI16/01873, PI16/01094, PI16/00501, PI16/00533, PI16/00381, PI16/00366, PI16/01522, PI16/01120, PI17/00764, PI17/01183, PI17/00855, PI17/01347, PI17/00525, PI17/01827, PI17/00532, PI17/00215, PI17/01441, PI17/00508, PI17/01732, PI17/00926, PI19/00957, PI19/00386, PI19/00309, PI19/01032, PI19/00576, PI19/00017, PI19/01226, PI19/00781, PI19/01560, PI19/01332, PI20/01802, PI20/00138, PI20/01532, PI20/00456, PI20/00339, PI20/00557, PI20/00886, and PI20/01158); the Especial Action Project entitled: Implementación y evaluación de una intervención intensiva sobre la actividad física Cohorte PREDIMED-Plus grant to JS-S; the European Research Council (Advanced Research Grant 2014–2019; agreement #340918) granted to ÁA-Gó; the Recercaixa (Number: 2013ACUP00194) grant to JS-S; grants from the Consejería de Salud de la Junta de Andalucía (PI0458/2013, PS0358/2016, and PI0137/2018); the PROMETEO/2017/017 and PROMETEO/2021/021 grants from the Generalitat Valenciana; Generalitat Valenciana AICO/2021/347 grant to JVid; the SEMERGEN grant; the Boosting young talent call grant program for the development of IISPV research projects 2019–2021 (Ref.: 2019/IISPV/03 grant to AD-L); the Societat Catalana d'Endocrinologia i Nutrició (SCEN) Clinical-Research Grant 2019 (IPs: JS-S and AD-L). Collaborative Nutrition and/or Obesity Project for Young Researchers 2019 supported by CIBEROBN entitled: Lifestyle Interventions and Chronic Kidney Disease: Inflammation, Oxidative Stress and Metabolomic Profile (LIKIDI study) grant to AD-L. Jordi Salas-Salvadó, gratefully acknowledges the financial support by ICREA under the ICREA Academia programme. CV-H receives a predoctoral grant from the Generalitat de Catalunya (2022 FI_B100108). None of the funding sources took part in the design, collection, analysis, interpretation of the data, or writing the report, or in the decision to submit the manuscript for publication.

## Conflict of interest

Author JS-S reported receiving research support from the Instituto de Salud Carlos III (ISCIII), Ministerio de Educación y Ciencia, Departament de Salut Pública de la Generalitat de Catalunya, the European Commission, the California Walnut Commission, Patrimonio Comunal Olivarero, La Morella Nuts, and Borges S.A; receiving consulting fees or travel expenses from California Walnut Commission, Eroski Foundation, Instituto Danone, Abbott Laboratories and Mundifarma, receiving non-financial support from Hojiblanca, Patrimonio Comunal Olivarero, and Almond Board of California; serving on the board of and receiving grant support through his institution from the International Nut and Dried Foundation and the Eroski Foundation; and grants and personal fees from Instituto Danone. Author ER reported receiving grants, personal fees, and non-financial support from the California Walnut Commission during the conduct of the study and grants, personal fees, non-financial support from Alexion; personal fees from Amarin; and non-financial support from the International Nut Council outside the submitted work. Author RE reported receiving grants from Instituto de Salud Carlos III and olive oil for the trial from Fundacion Patrimonio Comunal Olivarero/during the conduct of the study and personal fees from Brewers of Europe, Fundación Cerveza y Salud, Interprofesional del Aceite de Oliva, Instituto Cervantes, Pernaud Richar, Fundación Dieta Mediterránea, Wine and Culinary International Forum; non-financial support from Sociedad Española de Nutrición and Fundación Bosch y Gimpera; and grants from Uriach Laboratories outside the submitted work. Author XP reported receiving grants from ISCIII during the conduct of the study; receiving consulting fees from Sanofi Aventis, Amgen, and Abbott laboratories; receiving lecture personal fees from Esteve, Lacer and Rubio laboratories. The remaining authors declare that the research was conducted in the absence of any commercial or financial relationships that could be construed as a potential conflict of interest.

## Publisher's note

All claims expressed in this article are solely those of the authors and do not necessarily represent those of their affiliated organizations, or those of the publisher, the editors and the reviewers. Any product that may be evaluated in this article, or claim that may be made by its manufacturer, is not guaranteed or endorsed by the publisher.

## Abbreviations

BMI: Body Mass Index
CKD: Chronic Kidney Disease
CI: Confidence Interval
E: Energy
FFQ: Food Frequency Questionnaire
GFR: Glomerular Filtration Rate
MedDiet: Mediterranean Diet
MetS: Metabolic Syndrome
METS: Metabolic Equivalent Task
NEAP: Net Endogenous Acid Production
PRAL: Potential Renal Acid Load
PREDIMED: Prevención con Dieta Mediterránea
SCr: Serum Creatinine
CKD-EPI: Chronic Kidney Disease Epidemiology Collaboration equation for Caucasian individuals
OR: Odds Ratios
UACR: Urine Albumin/Creatinine Ratio.
