# Supplementary material for: Longitudinal association of dietary acid load with kidney function decline in an older adult population with metabolic syndrome
Source: Front Nutr. 2022 Sep 30;9:986190. doi: 10.3389/fnut.2022.986190 (PMC9563235; doi:10.3389/fnut.2022.986190)
Supplement: Supplementary file 1 [file Data_Sheet_1.docx]

**SUPPLEMETARY TABLES AND FIGURE LEGEND**

**Supplementary Table 1.** Consumption of food groups across tertiles of PRAL and NEAP at baseline of the study population with data on eGFR at 1-year of follow-up (n= 5874).

**Supplementary Table 2.** Baseline, one-year and changes in dietary intake of macronutrients and micronutrients of the study population with data on eGFR at 1-year of follow-up (n= 5874).

**Supplementary Table 3.** Baseline characteristics of the study population (n= 6,647) according to included or excluded individuals from the analysis of eGFR and UACR.

**Supplementary Table 4.** Multivariable-adjusted β-coefficients and 95% CI for 1-year changes in eGFR (ml/min/1.73m2) or 1-year changes in UACR (mg/g) across tertiles and per 1-SD increment of baseline PRAL and NEAP stratified by categories of eGFR and UACR.

**Supplementary Table 5.** Multivariable-adjusted β-coefficients and 95% CI of 1-year changes in eGFR (ml/min/1.73m2) or in UACR (mg/g) as well as adjusted OR and 95% CIs for ≥10% eGFR decline or ≥10% UACR increase by tertiles of baseline PRAL and NEAP.

**Supplementary Table 6.** Multivariable-adjusted OR (95% CIs) for ≥5% eGFR decline and ≥5% UACR increase by tertiles of baseline PRAL and NEAP and per 1-SD increment.

**Supplementary Figure 1.** Flow chart of the study population.

| **Supplementary Table 1. Consumption of food groups across tertiles of PRAL and NEAP at baseline of the study population with data on eGFR at 1-year of follow-up (n=** **5874).** | | | | | | | | |
| --- | --- | --- | --- | --- | --- | --- | --- | --- |
|  | **PRAL (mEq/d)** | | |  | **NEAP (mEq/d)** | | |  |
|  | **T1** | **T2** | **T3** |  | **T1** | **T2** | **T3** |  |
|  | n= 1958 | n= 1958 | n= 1958 | p-value | n= 1958 | n= 1958 | n= 1958 | p-value |
| Food groups |  |  |  |  |  |  |  |  |
| Fruits, g/day | 495.9 ± 233.9 | 333.3 ± 153.9 | 254.4 ± 140.0 | <0.01 | 480.4 ± 237.7 | 355.2 ± 161.9 | 248.0 ± 136.2 | <0.01 |
| Vegetables, g/day | 406.0 ± 152.4 | 313.2 ± 112.7 | 263.3 ± 101.8 | <0.01 | 395.1 ± 154.5 | 326.1 ± 117.5 | 261.3 ± 100.8 | <0.01 |
| Cereals, g/day | 134.6 ± 67.6 | 147.1 ± 75.4 | 170.6 ± 86.5 | <0.01 | 128.0 ± 65.1 | 151.9 ± 75.9 | 172.4 ± 86.2 | <0.01 |
| Whole grains, g/day | 88.5 ± 118.8 | 87.3 ± 129.8 | 81.2 ± 133.2 | 0.157 | 98.1 ± 124.3 | 93.7 ± 135.7 | 65.2 ± 119.3 | <0.01 |
| Refined grains, g/day | 117.4 ± 83.7 | 132.7 ± 91.2 | 165.5 ± 108.2 | <0.01 | 104.9 ± 77.6 | 135.8 ± 92.6 | 174.8 ± 105.7 | <0.01 |
| Legumes, g/day | 23.1 ± 13.1 | 20.0 ± 9.7 | 18.8 ± 9.7 | <0.01 | 22.3 ± 12.9 | 20.7 ± 10.1 | 18.9 ± 9.8 | <0.01 |
| Meat, fish and eggs, g/day | 248.6 ± 75.7 | 264.7 ± 74.1 | 308.0 ± 85.7 | <0.01 | 235.9 ± 73.2 | 274.9 ± 71.5 | 310.5 ± 84.9 | <0.01 |
| Milk and dairy products, g/day | 349.0 ± 210.9 | 342.9 ± 196.0 | 344.5 ± 194.2 | 0.61 | 344.4 ± 210.0 | 362.4 ± 201.2 | 329.7 ± 188.5 | <0.01 |
| Nuts, g/day | 17.3 ± 18.6 | 14.5 ± 16.3 | 13.1 ± 15.4 | <0.01 | 17.1 ± 18.9 | 15.0 ± 16.1 | 12.7 ± 15.1 | <0.01 |
| Olive oil, g/day | 40.2 ± 17.2 | 39.8 ± 16.8 | 40.8 ± 16.5 | 0.14 | 39.2 ± 17.1 | 40.3 ± 16.8 | 41.3 ± 16.6 | <0.01 |
| Other fats, g/day | 2.6 ± 6.1 | 3.1 ± 6.6 | 3.1 ± 6.8 | 0.07 | 2.6 ± 5.9 | 3.0 ± 6.5 | 3.2 ± 7.0 | 0.02 |
| Sugar and sweetened products, g/day | 46.9 ± 64.6 | 53.7 ± 71.6 | 65.3 ± 86.5 | <0.01 | 47.3 ± 67.0 | 54.5 ± 75.2 | 64.0 ± 81.6 | <0.01 |
| Alcohol, g/day | 11.3 ± 15.8 | 11.3 ± 15.4 | 11.1 ± 14.4 | 0.92 | 10.5 ± 15.2 | 11.3 ± 15.5 | 11.8 ± 14.8 | 0.04 |
| **Abbreviations:**  *eGFR*, Estimated glomerular filtration rate; *NEAP*, Net Endogenous Acid Production; *T,* tertile; *PRAL*, Potential Renal Acid Load. Values are presented as means ± standard deviations. P-value was calculated by one-way analysis of variance test. | | | | | | | | |

| **Supplementary Table 2. Baseline, one-year, and changes in dietary intake of macronutrients and micronutrients of the study population with data on eGFR at 1-year of follow-up (n=** **5874).** | | | | | |
| --- | --- | --- | --- | --- | --- |
|  | | **Baseline** | **1-year** | **Change** |  |
|  | | **n= 5874** | **n=5675** | **n=5675** | P-value |
| Carbohydrate intake, g/day | | 240.9 ± 72.5 | 209.1 ± 0.8 | -31.6 ± 0.9 | <0.001 |
| Protein intake, g/day | | 97.7 ± 22.0 | 95.4 ± 0.3 | -2.2 ± 0.3 | <0.001 |
| Fat intake, g/day | | 104.2 ± 28.3 | 103.7 ± 0.3 | -0.7 ± 0.4 | 0.07 |
|  | Monounsaturated fatty acids, g/day | 54.0 ± 16.1 | 58.7 ± 0.2 | 4.5 ± 0.2 | <0.001 |
|  | Polyunsaturated fatty acids, g/day | 18.0 ± 6.6 | 19.0 ± 0.08 | 1.0 ± 0.1 | <0.001 |
|  | Saturated fatty acids intake, g/day | 26.3 ± 8.4 | 22.8 ± 0.1 | -3.5 ± 0.11 | <0.001 |
| Fiber intake, g/day | | 26.1 ± 8.8 | 29.8 ± 0.1 | 3.7 ± 0.1 | <0.001 |
| Phosphorus intake, mg/day | | 1759.11 ± 419.9 | 1780.2 ± 5.5 | 22.5 ± 5.6 | <0.001 |
| Potassium intake, mg/day | | 4477.0 ± 1079.6 | 4673.6 ± 13.6 | 201.3 ± 14.5 | <0.001 |
| Magnesium intake, mg/day | | 420.4 ± 108.2 | 453.9 ± 1.5 | 420.0 ± 1.4 | <0.001 |
| Calcium intake, mg/day | | 1034.0 ± 347.0 | 992.9 ± 4.2 | -40.1 ± 4.4 | <0.001 |
| Sodium intake, mg/day | | 2430.0 ± 774.8 | 2081.1 ± 8.9 | -345.8 ± 10.5 | <0.001 |
| **Abbreviations:**  *eGFR*, Estimated glomerular filtration rate. Values are presented as means ± standard deviations. P-value was calculated by T-test. | | | | | |

| **Supplementary Table 3. Baseline characteristics of the study population (n=** **6,647) according to included or excluded individuals from the analysis of eGFR and UACR.** | | | | | | |
| --- | --- | --- | --- | --- | --- | --- |
|  | **eGFR** | | | **UACR** | | |
|  | **Excluded** | **Included** |  | **Excluded** | **Included** |  |
|  | n= 773 | n= 5874 | p-value | n= 3008 | n= 3639 | p-value |
| PRAL, mEq/day | -5.2 ± 16.1 | -5.4 ± 15.6 | 0.66 | -6.0 ± 16.5 | -5.0 ± 14.9 | 0.01 |
| NEAP, mEq/day | 37.1 ± 8.6 | 37.0 ± 8.1 | 0.66 | 36.7 ± 8.4 | 37.2 ± 8.0 | 0.01 |
| Age, years | 64.8 ± 5.0 | 65.0 ± 4.9 | 0.27 | 65.1 ± 4.9 | 64.9 ± 5.0 | 0.04 |
| Women, % (n) | 400 (51.8) | 2818 (48.0) | 0.05 | 54.7 (1646) | 43.2 (1572) | <0.01 |
| Intervention group, % (n) | 399 (51.6) | 2901 (49.4) | 0.244 | 51.2 (1541) | 48.3 (1759) | 0.02 |
| BMI, kg/m^2^ | 32.8 ± 3.5 | 32.5 ± 3.4 | 0.04 | 32.6 ± 3.5 | 32.5 ± 3.4 | 0.11 |
| PA, METS/min/week | 1972.6 ± 1842.9 | 2528.0 ± 2350.4 | <0.01 | 2326.8 ± 2221.1 | 2576.4 ± 2364.5 | <0.01 |
| Smoking status, % (n) |  |  | 0.24 |  |  | <0.01 |
| Never smoked | 343 (44.4) | 2605 (44.4) |  | 47.5 (1430) | 41.7 (1518) |  |
| Former smoker | 348 (45.0) | 2528 (43.0) |  | 40.5 (1219) | 45.5 (1657) |  |
| Current smoker | 82 (10.6) | 741 (12.6) |  | 11.9 (359) | 12.8 (464) |  |
| Education level, % (n) |  |  | 0.10 |  |  | 0.26 |
| Primary education | 379 (49.0) | 2891 (49.2) |  | 50.3 (1513) | 48.3 (1757) |  |
| Secondary education | 204 (26.4) | 1714 (29.2) |  | 28.3 (850) | 29.4 (1068) |  |
| College/University | 190 (24.6) | 1269 (21.6) |  | 21.4 (645) | 22.4 (814) |  |
| Creatinine | 0.8 ± 0.2 | 0.8 ± 0.2 | 0.18 | 0.8 ± 0.22 | 0.8 ± 0.2 | 0.731 |
| eGFR, ml/min/1.73m^2^ | 84.6 ± 14.6 | 84.2 ± 14.0 | 0.47 | 83.1 ± 14.5 | 85.2 ±13.6 | <0.01 |
| UACR, mg/g | 21.7 ± 68.6 | 16.8 ± 48.9 | 0.05 | 18.1 ± 59.4 | 17.1 ± 48.7 | 0.58 |
| CKD, % (n) | 40 (5.8) | 387 (6.6) | 0.42 | 7.3 (214) | 5.9 (213) | 0.02 |
| Type 2 diabetes, % (n) | 250 (32.3) | 1797 (30.6) | 0.32 | 32.7 (983) | 29.2 (1064) | 0.02 |
| Hypertension, % (n) | 642 (83.1) | 4941 (84.1) | 0.45 | 83.6 (2515) | 84.3 (3068) | 0.44 |
| Hypercholesterolemia, % (n) | 553 (71.5) | 4096 (69.7) | 0.30 | 72.4 (2178) | 67.9 (2471) | <0.01 |
| Medication use, % (n) |  |  |  |  |  |  |
| Lipid-lowering drugs | 416 (53.8) | 3042 (51.8) | 0.29 | 53.9 (1,622) | 50.5 (1836) | 0.01 |
| Oral blood glucose-lowering drugs | 216 (28.0) | 1528 (26.0) | 0.25 | 28.2 (849) | 24.6 (895) | <0.01 |
| Insulin treatment | 40 (5.17) | 239 (4.1) | 0.15 | 4.5 (136) | 3.9 (143) | 0.23 |
| Antihypertensive drugs | 583 (75.4) | 4625 (78.7) | 0.04 | 77.3 (2324) | 79.3 (2884) | 0.05 |
| ARBs | 292 (37.8) | 2131 (36.3) | 0.42 | 38.0 (1143) | 35.2 (1280) | 0.02 |
| ACEis | 217 (28.1) | 1775 (30.2) | 0.22 | 28.0 (843) | 31.6 (1149) | <0.01 |
| Dietary assessment |  |  |  |  |  |  |
| erMedDiet score, 17-points | 8.6 ± 2.7 | 8.5 ± 2.7 | 0.39 | 8.6 ± 2.7 | 8.5 ± 2.7 | 0.12 |
| Energy intake, kcal/d | 2325.2 ± 569.3 | 2370.5 ± 548.9 | 0.03 | 2343.6 ± 553.7 | 2383.2 ± 549.1 | 0.01 |
| Protein intake, % energy | 17.1 ± 2.8 | 16.7 ± 2.8 | <0.01 | 17.2 ± 2.9 | 16.4 ± 2.7 | <0.01 |
| Fat intake, % energy | 39.0 ± 6.9 | 39.6 ± 6.5 | 0.02 | 39.3 ± 6.6 | 39.8 ± 6.5 | <0.01 |
| Carbohydrate intake, % energy | 41.1 ± 7.1 | 40.5 ± 6.8 | 0.02 | 40.9 ± 6.9 | 40.3 ± 6.8 | <0.01 |
| Fiber intake, g/day | 26.1 ± 8.9 | 26.1 ± 8.8 | 0.92 | 26.9 ± 9.0 | 25.5 ± 8.5 | <0.01 |
| Potassium intake, mg/day | 4465.2 ± 1078.5 | 447.0 ± 1079.6 | 0.78 | 4587.0 ± 1119.8 | 4383.5 ± 1036.0 | <0.01 |
| Calcium intake, mg/day | 1037.0 ± 332.7 | 1034.0 ± 347.0 | 0.82 | 1059.8 ± 346.6 | 1013.4 ± 342.8 | <0.01 |
| Magnesium intake, mg/day | 420.5 ± 110.8 | 420.4 ± 108.2 | 0.98 | 429.4 ± 111.00 | 412.9 ± 105.9 | <0.01 |
| Phosphorus intake, mg/day | 1761.3 ± 429.4 | 1759.1 ± 419.9 | 0.89 | 1799.6 ± 428.8 | 1726.1 ± 411.5 | <0.01 |
| Sodium intake, mg/day | 2386.9 ± 798.4 | 2430.0 ± 774.8 | 0.15 | 2419.3 ± 778.1 | 2429.6 ± 777.4 | 0.59 |
| **Abbreviations:** *ACEis*, Angiotensin-Converting Enzyme Inhibitors; *ARBs*, Angiotensin II receptor blockers; *eGFR*, Estimated Glomerular Filtration Rate; *erMedDiet*, energy-restricted Mediterranean diet, *NEAP*, Net Endogenous Acid Production; *T*, tertile; *BMI*, Body Mass Index; *PRAL*, Potential Renal Acid Load; *PA*, Physical activity; *eGFR*, estimated Glomerular Filtration Rate; *CKD*, Chronic Kidney Disease (eGFR<60 ml/min/1.73m^2^); *UACR*, Urine Albumin/Creatinine Ratio. Values are presented as percentages (n) for categorical variables and means ± standard deviations for continuous variables. P-value was calculated by chi-square or one-way analysis of variance test for categorical and continuous variables, respectively. | | | | | | |

| **Supplementary Table 4. Multivariable-adjusted β-coefficients and 95% CI for 1-year changes in eGFR (ml/min/1.73m^2^) or 1-year changes in UACR (mg/g) across tertiles and per 1-SD increment of baseline PRAL and NEAP stratified by categories of eGFR and UACR.** | | | | | | |
| --- | --- | --- | --- | --- | --- | --- |
| **PRAL** | | | | | | |
|  | | **T1** | **T2** | **T3** | **p for Trend** | **Continuous**  (1 SD⁑) |
| Δ in eGFR, ml/min/1.73m^2^ | |  | | |  |  |
| eGFR categories | |  |  |  |  |  |
|  | **≥ 90 ml/min/1.73m^2^** |  |  |  |  |  |
|  | No. | n= 838 | n= 838 | n= 837 |  | n= 2513 |
|  | Model 1 | 0 (Ref.) | -0.18 (-0.88 to 0.51) | -0.50 (-1.18 to 0.18) | 0.151 | -0.22 (-0.48 to 0.03) |
|  | Model 2 | 0 (Ref.) | -0.26 (-0.98 to 0.45) | -0.70 (-1.41 to 0.01) | 0.056 | -0.28 (-0.56 to -0.01)* |
|  | **60 – 90 ml/min/1.73m^2^** |  | | |  |  |
|  | No. | n= 992 | n= 991 | n= 991 |  | n=2974 |
|  | Model 1 | 0 (Ref.) | 0.05 (-0.74 to 0.84) | -0.31 (-1.13 to 0.51) | 0.463 | -0.12 (-0.46 to 0.21) |
|  | Model 2 | 0 (Ref.) | 0.04 (-0.75 to 0.84) | -0.43 (-1.28 to 0.42) | 0.331 | -0.18 (-0.53 to 0.17) |
|  | **< 60 ml/min/1.73m^2^** |  | | |  |  |
|  | No. | n= 129 | n= 129 | n= 129 |  | n= 387 |
|  | Model 1 | 0 (Ref.) | 0.03 (-2.67 to 2.74) | -0.77 (-3.30 to 1.77) | 0.568 | -0.40 (-1.35 to 0.56) |
|  | Model 2 | 0 (Ref.) | 0.80 (-1.95 to 3.57) | -0.52 (-3.24 to 2.20) | 0.736 | -0.24 (-1.30 to 0.81) |
| Δ in UACR, mg/g | |  | | |  |  |
| UACR categories | |  | | |  |  |
|  | **< 30 mg/g** |  | | |  |  |
|  | No. | n= 1099 | n= 1099 | n= 1098 |  | n= 3296 |
|  | Model 1 | 0 (Ref.) | -0.13 (-2.06 to 1.80) | 0.07 (-1.56 to 1.70) | 0.935 | 0.07 (-0.55 to 0.70) |
|  | Model 2 | 0 (Ref.) | -0.29 (-2.28 to 1.70) | -0.19 (-2.06 to 1.68) | 0.835 | -0.01 (-0.71 to 0.69) |
|  | **≥ 30 mg/g** |  | | |  |  |
|  | No. | n= 115 | n= 114 | n= 114 |  | n=343 |
|  | Model 1 | 0 (Ref.) | -11.17 (-38.85 to 6.50) | -24.54 (-50.53 to 1.46) | 0.072 | -8.54 (-18.15 to 1.07) |
|  | Model 2 | 0 (Ref.) | -8.52 (-36.46 to 19.41) | -30.06 (-58.88 to -1.24)* | 0.055 | -10.71 (-22.26 to 0.84) |
| **NEAP** | | | | | | |
| Δ in eGFR, ml/min/1.73m^2^ | |  |  |  |  |  |
| eGFR categories | |  |  |  |  |  |
|  | **≥ 90 ml/min/1.73m^2^** |  |  |  |  |  |
|  | No. | n= 838 | n= 838 | n= 837 |  | n= 2513 |
|  | Model 1 | 0 (Ref.) | -0.28 (-0.98 to 0.41) | -0.24 (-0.92 to 0.43) | 0.494 | -0.20 (-0.45 to 0.06) |
|  | Model 2 | 0 (Ref.) | -0.36 (-1.07 to 0.35) | -0.47 (-1.18 to 0.25) | 0.207 | -0.31 (-0.58 to -0.03)* |
|  | **60 – 90 ml/min/1.73m^2^** |  | | |  |  |
|  | No. | n= 992 | n= 991 | n= 991 |  | n=2974 |
|  | Model 1 | 0 (Ref.) | -0.19 (-0.98 to 0.60) | -0.40 (-1.23 to 0.43) | 0.344 | -0.15 (-0.49 to 0.19) |
|  | Model 2 | 0 (Ref.) | -0.14 (-0.94 to 0.66) | -0.46 (-1.32 to 0.40) | 0.287 | -0.18 (-0.54 to 0.18) |
|  | **< 60 ml/min/1.73m^2^** |  | | |  |  |
|  | No. | n= 129 | n= 129 | n= 129 |  | n= 387 |
|  | Model 1 | 0 (Ref.) | -1.15 (-3.81 to 1.50) | -1.10 (-3.73 to 1.54) | 0.420 | -0.60 (-1.58 to 0.38) |
|  | Model 2 | 0 (Ref.) | -0.55 (-3.29 to 2.18) | -1.07 (-3.85 to 1.71) | 0.448 | -0.49 (-1.56 to 0.58) |
| Δ in UACR, mg/g | |  |  |  |  |  |
| UACR categories | |  |  |  |  |  |
|  | **< 30 mg/g** |  |  |  |  |  |
|  | No. | n= 1099 | n= 1099 | n= 1098 |  | n= 3296 |
|  | Model 1 | 0 (Ref.) | -0.58 (-2.43 to 1.28) | 0.11 (-1.66 to 1.89) | 0.876 | 0.10 (-0.55 to 0.75) |
|  | Model 2 | 0 (Ref.) | -0.64 (-2.30 to 1.02) | -0.04 (-1.77 to 1.69) | 0.999 | 0.04 (-0.66 to 0.74) |
|  | **≥ 30 mg/g** |  |  |  |  |  |
|  | No. | n= 115 | n= 114 | n= 114 |  | n=343 |
|  | Model 1 | 0 (Ref.) | -9.54 (-37.37 to 18.29) | -27.10 (-37.37 to 18.29) | 0.149 | -9.56 (-19.69 to 0.56) |
|  | Model 2 | 0 (Ref.) | -11.65 (-39.66 to 6.35) | -29.71 (-61.06 to 1.65) | 0.087 | -10.39 (-22.44 to 1.66) |
| **Abbreviations**: *eGFR*, Estimated glomerular filtration rate; *NEAP*, Net Endogenous Acid Production; *T*, tertile; *PRAL*, Potential Renal Acid Load; *UACR*, Urine albumin/creatinine ratio. Model 1: adjusted for age (years), sex and baseline eGFR or baseline UACR (in continuous, depending on the main outcome). Model 2: additionally adjusted for participating center (categorized into quartiles by number of participants), intervention group (treatment/control), body mass index (kg/m^2^), smoking habits (never, current or former smoker), educational level (primary, secondary education or graduate), leisure-time physical activity (METS/min/week in tertiles), diabetes prevalence (yes/no), hypertension prevalence (yes/no) and hypercholesterolemia prevalence (yes/no), ARBs (yes/no), ACEis (yes/no), Mediterranean diet adherence (high/low adherence), energy intake (kcal/day in tertiles), sodium intake (mg/g in tertiles) and high leukocytes levels (yes/no). *p-value < 0.05. ⁑One SD= 15.6 mEq/d in PRAL and 8.1 mEq/d in NEAP. | | | | | | |

| **Supplementary Table 5. Multivariable-adjusted β-coefficients and 95% CI of 1-year changes in eGFR (ml/min/1.73m2) or in UACR (mg/g) as well as adjusted OR and 95% CIs for ≥10% eGFR decline or ≥10% UACR increase by tertiles of baseline PRAL and NEAP.** | | | | |
| --- | --- | --- | --- | --- |
| **PRAL (mEq/d)** | | | | |
|  | **T1** | **T2** | **T3** | **p for Trend** |
| **Δ in eGFR, ml/min/1.73m^2^** | n= 1958 | n= 1958 | n= 1958 |  |
| Model 2 | 0 (Ref.) | -0.14 (-0.68 to 0.39) | -0.60 (-1.16 to -0.04)* | 0.037 |
| **eGFR decline ≥10%** | n= 296; %= 15.1 | n= 304; %= 15.5 | n= 346; %= 17.7 |  |
| Model 2 | 1 (Ref.) | 1.04 (0.87 to 1.25) | 1.25 (1.05 to 1.50)* | 0.017 |
| **Δ in UACR, mg/g** | n= 1213 | n= 1213 | n= 1213 |  |
| Model 2 | 0 (Ref.) | -1.53 (-4.75 to 1.69) | -2.83 (-6.19 to 0.57) | 0.100 |
| **UACR increase ≥5%** | n= 539; %= 44.4 | n= 547; %= 45.1 | n= 597; %= 49.2 |  |
| Model 2 | 1 (Ref.) | 1.04 (0.88 to 1.23) | 1.23 (1.03 to 1.46)* | 0.021 |
| **NEAP (mEq/d)** | | | | |
| **Δ in eGFR, ml/min/1.73m^2^** | n= 1958 | n= 1958 | n= 1958 |  |
| Model 2 | 0 (Ref.) | -0.27 (-0.80 to 0.26) | -0.51 (-1.08 to 0.06) | 0.080 |
| **eGFR decline ≥10%** | n= 297; %= 15.2 | n= 312; %= 15.9 | n= 337; %= 17.2 |  |
| Model 2 | 1 (Ref.) | 1.06 (0.89 to 1.26) | 1.21 (1.01 to 1.46)* | 0.044 |
| **Δ in UACR, mg/g** | n= 1213 | n= 1213 | n= 1213 |  |
| Model 2 | 0 (Ref.) | -0.72 (-3.77 to 2.33) | -2.23 (-5.58 to 1.11) | 0.184 |
| **UACR increase ≥5%** | n= 550; %= 45.3 | n= 539; %= 44.4 | n= 594; %= 49.0 |  |
| Model 2 | 1 (Ref.) | 0.97 (0.82 to 1.14) | 1.18 (0.99 to 1.41)* | 0.053 |
| **Abbreviations**: *eGFR*, Estimated glomerular filtration rate; *NEAP*, Net Endogenous Acid Production; *T*, tertile; *PRAL*, Potential Renal Acid Load; *UACR*, Urine albumin/creatinine ratio. All models were adjusted for age (years), sex, baseline eGFR or baseline UACR (in continuous, in logistic regression models depending on the main outcome), for participating center (categorized into quartiles by number of participants), intervention group (treatment/control), body mass index (kg/m^2^), smoking habits (never, current or former smoker), educational level (primary, secondary education or graduate), leisure-time physical activity (METS/min/week in tertiles), diabetes prevalence (yes/no), hypertension prevalence (yes/no) and hypercholesterolemia prevalence (yes/no), ARBs (yes/no), ACEis (yes/no), Mediterranean diet adherence (high/low, ≥/< 12 points), energy intake (kcal/day in tertiles), sodium intake (mg/g in tertiles) and high leukocytes levels (yes/no). *p-value < 0.05. | | | | |

| **Supplementary Table 6. Multivariable-adjusted OR (95% CIs) for ≥5% eGFR decline and ≥5% UACR increase by tertiles of baseline PRAL and NEAP and per 1-SD increment.** | | | | | |
| --- | --- | --- | --- | --- | --- |
| PRAL | | | | | |
|  | **T1** | **T2** | **T3** | **p for Trend** | **Continuous**  (1 SD⁑) |
| **eGFR decline ≥5%** |  |  |  |  |  |
| No. (%) | 527 (26.9) | 547 (27.9) | 578 (29.5) |  | 1652 (28.1) |
| Model 1 | 1 (Ref.) | 1.05 (0.92 to 1.21) | 1.16 (1.01 to 1.34)* | 0.037 | 1.08 (1.02 to 1.14)* |
| Model 2 | 1 (Ref.) | 1.06 (0.92 to 1.23) | 1.20 (1.04 to 1.40)* | 0.017 | 1.10 (1.03 to 1.17)* |
| **UACR increase ≥5%** |  |  |  |  |  |
| No. (%) | 580 (47.8) | 583 (48.1) | 633 (52.2) |  | 1796 (49.4) |
| Model 1 | 1 (Ref.) | 1.02 (0.87 to 1.19) | 1.21 (1.03 to 1.43)* | 0.023 | 1.06 (0.99 to 1.13) |
| Model 2 | 1 (Ref.) | 1.02 (0.86 to 1.20) | 1.19 (1.00 to 1.42)* | 0.054 | 1.05 (0.98 to 1.13) |
| NEAP | | | | | |
| **eGFR decline ≥5%** |  |  |  |  |  |
| No. (%) | 532 (27.2) | 546 (27.9) | 574 (29.3) |  | 1652 (28.1) |
| Model 1 | 1 (Ref.) | 1.03 (0.90 to 1.19) | 1.14 (0.99 to 1.32) | 0.062 | 1.08 (1.02 to 1.14)* |
| Model 2 | 1 (Ref.) | 1.04 (0.90 to 1.20) | 1.18 (1.02 to 1.38)* | 0.029 | 1.10 (1.03 to 1.17)* |
| **UACR increase ≥5%** |  |  |  |  |  |
| No. (%) | 589 (48.56) | 581 (47.90) | 626 (51.61) |  | 1796 (49.4) |
| Model 1 | 1 (Ref.) | 0.98 (0.83 to 1.15) | 1.16 (0.98 to 1.36) | 0.076 | 1.05 (0.98 to 1.12) |
| Model 2 | 1 (Ref.) | 0.96 (0.82 to 1.14) | 1.13 (0.95 to 1.35) | 0.148 | 1.04 (0.97 to 1.12) |
| **Abbreviations**: *eGFR*, Estimated glomerular filtration rate; *NEAP*, Net Endogenous Acid Production; *T*, tertile; *PRAL*, Potential Renal Acid Load; *UACR*, Urine albumin/creatinine ratio. Model 1: adjusted for age (years) and sex. Model 2: additionally adjusted for participating center (categorized into quartiles by number of participants), intervention group (treatment/control), body mass index (kg/m^2^), smoking habits (never, current or former smoker), educational level (primary, secondary education or graduate), leisure-time physical activity (METS/min/week in tertiles), diabetes prevalence (yes/no), hypertension prevalence (yes/no) and hypercholesterolemia prevalence (yes/no), ARBs (yes/no), ACEis (yes/no), Mediterranean diet adherence (high/low adherence), energy intake (kcal/day in tertiles), sodium intake (mg/g in tertiles) and high leukocytes levels (yes/no). *p-value < 0.05. ⁑One SD= 15.6 mEq/d in PRAL and 8.1 mEq/d in NEAP. | | | | | |


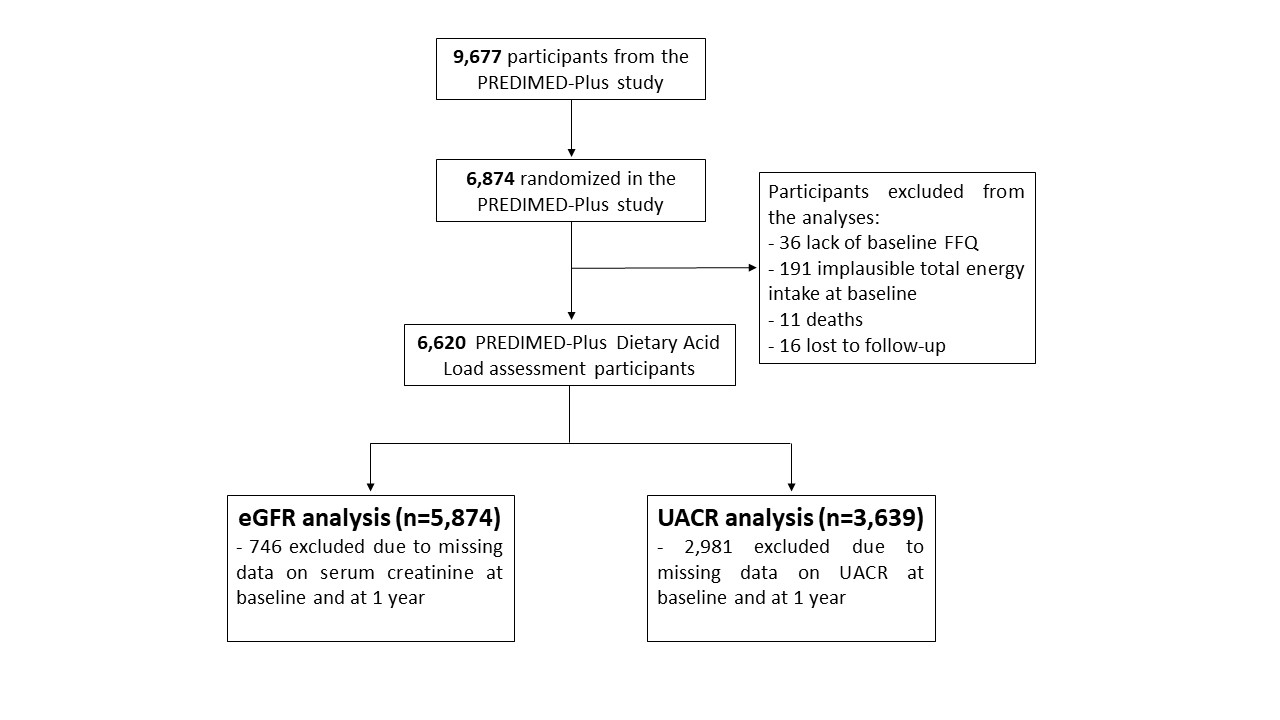


**Supplementary Figure 1. Flow chart of the study population.**
